# The Hydrophobicity of Lignocellulosic Fiber Network Can Be Enhanced with Suberin Fatty Acids

**DOI:** 10.3390/molecules24234391

**Published:** 2019-12-01

**Authors:** Risto I. Korpinen, Petri Kilpeläinen, Tytti Sarjala, Maristiina Nurmi, Pauliina Saloranta, Thomas Holmbom, Hanna Koivula, Kirsi S. Mikkonen, Stefan Willför, Pekka T. Saranpää

**Affiliations:** 1 Production Systems, Natural Resources Institute Finland, Latokartanonkaari 9, FI-00790 Helsinki, Finland; petri.kilpelainen@luke.fi (P.K.); tytti.sarjala@luke.fi (T.S.); pekka.saranpaa@luke.fi (P.T.S.); 2 Laboratory of Paper Coating and Converting, Center for Functional Materials, Åbo Akademi University, Porthaninkatu 3, FI-20500 Turku, Finland; manurmi@abo.fi (M.N.); Pauliina@metgen.com (P.S.); 3 Oy Separation Research Ab, Porthaninkatu 3, FI-20500 Turku, Finland; tholmbom@sepres.com; 4 Department of Food and Nutrition, Faculty of Agriculture and Forestry, University of Helsinki, Agnes Sjöbergin katu 2, FI-00014 Helsinki, Finland; hanna.m.koivula@helsinki.fi (H.K.); kirsi.s.mikkonen@helsinki.fi (K.S.M.); 5 Laboratory of Wood and Paper Chemistry, Johan Gadolin Process Chemistry Centre, Åbo Akademi University, Porthaninkatu 3, FI-20500 Turku, Finland; stefan.willfor@abo.fi

**Keywords:** Birch outer bark, suberin fatty acids, lignocellulosic fiber network, hydrophobicity, contact angle, scanning electron microscope, time-of-flight secondary ion mass spectrometry

## Abstract

Suberin fatty acids were extracted from outer bark of Silver birch (*Betula pendula* Roth.) using an isopropanolic sodium hydroxide solution. Laboratory sheets composed of lignocellulosic fiber networks were prepared from unbleached and unrefined softwood kraft pulp and further impregnated with suberin fatty acid monomers and cured with maleic anhydride in ethanol solution. The treatment resulted in hydrophobic surfaces, in which the contact angles remained over 120 degrees during the entire measurement. The fiber network also retained its water vapor permeability and enhanced fiber–fiber bonding resulted in improved tensile strength of the sheets. Scanning electron microscopy (SEM) images revealed that the curing agent, together with suberin fatty acids, was evenly distributed on the fiber surfaces and smoothing occurred over the wrinkled microfibrillar structure. High concentrations of the curing agent resulted in globular structures containing betulinol derivates as revealed with time-of-flight secondary ion mass spectrometry (ToF-SIMS). Also, the larger amount of suberin fatty acid monomers slightly impaired the optical properties of sheets.

## 1. Introduction

Synthetic polymers such as polyethylene, polyvinylchloride or polystyrene have excellent material properties that can be used in many applications. However, since they are not biodegradable, they accumulate in landfills. If not disposed properly, they may end up in the oceans, where they persist [1]. Plastics degrade further to microplastics that can harm marine organisms [2]. Polymer durability is usually a desired property and it is the actual littering which causes the main problem. Therefore, there is a need for the development of sustainable polymers and materials that can be used as barriers, coatings or films. The new products should biodegrade in nature, or, more preferably, be compostable in controlled conditions.

Cellulose fibers, which are widely used for papermaking, paperboard, cardboard, textiles and specialty chemicals, can be recycled after use and utilized for several cycles [3]. Before combusting or composting, the fibers can be utilized for ethanol production, nanocellulose applications or biobased chemicals according the cascading principle. Developed countries have significantly reduced the use of paper in printing but fibers are increasingly used to make packaging materials. Wood is also a preferable source for textile fibers since trees can be grown without the use of large amounts of water, fertilizers and pesticides, which are needed in cotton production. Cotton production demands large land areas that could be used for food production instead [4]. In 2013–2014, cotton was harvested on ca. 32,430,000 hectares corresponding to 2.3% of the world’s arable land area [5]. Novel methods have been developed in the production of textiles from kraft and dissolving pulp [6]. Also, ionic liquids have been used [7].

Textiles and packaging materials from natural biodegradable materials need functional properties such as water repellency. Paper and board as such will disintegrate gradually if wet. To prevent this, cardboard is traditionally laminated with polyethylene films. As an alternative, the lignocellulosic surfaces can be treated with natural substances derived from bark which form structures that repel water [8,9].

Birch wood (*Betula pendula* Roth. and *Betula pubescens* Ehrh.) is a valuable raw material for the furniture industry and veneer manufacturing in the Nordic countries. In 2017, the Finnish forest industry alone consumed approximately 14.3 million m^3^ hardwood logs, mainly birch [10]. A volume of 2.7 m^3^ birch roundwood contains on average 28.6 kg oven dry outer bark (10.6 kg o.d. m^−3^) [11]. Based on these figures it can be estimated that the amount of oven dry outer bark produced is over 150,000 tonnes annually in Finland. Currently, it is solely used for producing bioenergy.

Birch outer bark is rich in valuable biochemicals like the naturally occurring biopolyester suberin and the triterpenoids betulinol and lupeol [12,13,14,15,16]. The content of betulinol and lupeol in outer bark varies between 30%–35% and the content of suberin can be up to 40%–50% [13,17]. Birch bark containing both inner and outer bark from a pulp mill has been reported to contain 5.9% of suberin [18]. Suberin is a complex polymer, built by long-chain bifunctional ω-hydroxyacids and α,ω-diacids that are both C18 midchain modified and saturated, long-chain monofunctional fatty acids, and fatty alcohols which are interlinked by ester-bonds to glycerol. When suberin is depolymerized, the main components are long-chain aliphatic acids, typically 80%–90% of depolymerisates [19]. Suberin fatty acids are covalently linked through esterification to ferulic acid and neighboring lignin-like polyaromatics [12,19,20,21]. Suberin is believed to form partly orderly lamellar structures [19,22].

Due to its complex structure, suberin extraction requires special conditions. It has been extracted as fatty acid salts by alkaline alcohol reagents, such as ethanol with sodium hydroxide [11,12,23,24,25]. Ionic liquids such as 1-ethyl-3-methylimidazolium hexanoate, cholinium hexanoate, cholinium octanoate, and cholinium decanoate have been applied on extraction of suberin from cork [26,27,28,29]. The extraction yield of suberin varied between 30.6% and 67.2%. The maximum yield of suberin with alkaline methanolysis has been reported to be approximately 55% [20]. Due to the problems related to the recovery of ionic liquid, we focused on conventional alkaline alcoholic extraction of suberin fatty acids (SFAs) because alcohols are relatively easy to recover due to their high vapor pressure.

Our goal is to develop a resource-efficient method to extract suberin fatty acids from birch bark and investigate the possibility of using them as hydrophobic coatings for lignocellulosic fibers. We are aiming to replace fossil raw materials with completely sustainable raw materials to create water-repellent and functional surface coatings for cellulose-based packaging materials and textiles.

## 2. Results

### 2.1. SFA and Betulinol Fractions

**Figure 1:**
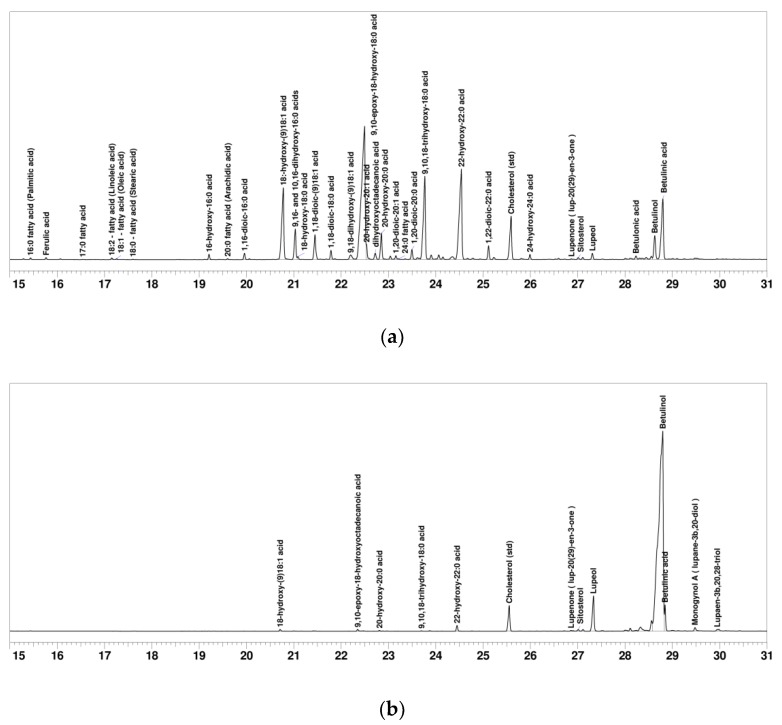
Gas chromatograms of suberin fatty acid fraction (**a**) and betulinol fraction (**b**) from birch outer bark.

The amount of SFAs and betulinol in outer bark was at the same level as reported earlier [13], i.e., 29.4% and 27.9% (o.d.), respectively. SFAs were mainly 18 and 22 carbon fatty acids (Figure 1a and Table 1). Major fatty acids were 9,10-epoxy-18-hydroxy-18:0 acid, 22-hydroxy-22 acid, 9,10,18-trihydroxy-18:0 acid and 18:-hydroxy-(9) 18:1 acid. The suberin fraction also contained some betulinol and betulinic acid (Figure 1b and Table 1). The fatty acid composition was similar to those reported for suberized tissues of birch outer bark and oak bark [22,23].

### 2.2. Elemental Analysis of SFA and Betulinol Fractions

Table 2 shows the results of the inorganic elemental analysis of the obtained fractions after extraction and subsequent precipitations. The major inorganic element in the SFA fraction was sulfur. SFA soaps were converted to acid form with sulfuric acid and some of the formed sulfates were most probably attached to the precipitated material. The average ash content of birch bark from a pulp mill has been reported to be 2.9% [18]. The amounts of Cu, Zn, Ni, Cr and Pb were lower in the suberin and betulinol fractions compared to earlier reports [18].

### 2.3. The Effect of SFA Curing Agent Applied to the Laboratory Sheets (Lignocellulosic Fiber Network)

#### 2.3.1. Basic Properties

The grammage of the laboratory sheets correlated to the amount of curing agent (Table 3). The grammage of the reference laboratory sheets and the laboratory sheets that did not have any suberin was on average 57.1 g m^−2^. However, grammage of laboratory sheets increased less than expected. If the amount of curing agent was increased from 10 to 30 g m^−2^, the grammage of sheets increased only to 62.3 and 69.8 g m^−2^. The calculated laboratory sheet grammage after SFA curing agent addition, assuming complete absorption, should have been 67.1, 77.1 and 87.1 g m^−2^. Only half of the curing agent was absorbed in the fiber network. The more curing agent that was added, the less it was absorbed by the fibers, from 52.2% to 42.5% of the calculated amount. Some visible vapor was observed during the heat treatment. Also, the aluminum foils contained colorful stains after curing. This means that most probably some of the curing agent was evaporated and also attached to the aluminum foil supporting the sheets.

The thickness of the laboratory sheets increased in correlation with the amount of curing agent (Table 3). The reference sheet and the laboratory sheet without curing agent had similar sheet thickness of 182 µm. With 10 g m^−2^ curing agent the thickness increased to 190 µm. When 20 and 30 g m^−2^ treatment was performed, the thickness increased further to 205 and 207 µm, respectively. The maximum increase in thickness was 13.7% as compared to the reference sheet. Table 3 shows how the fibers become thicker with SFA curing treatment.

Tensile index increased as a function of increased amount of SFA curing agent on the surface from 11.5 Nm g^−1^ to 16.7 Nm g^−1^ (Table 3). The values are relatively low but the pulp was unrefined to obtain a porous sheet structure. Unlike grammage, thickness, density and tensile index, the tear index decreased as more curing agent was applied on the laboratory sheets. The tear index decreased from 5.7 mNm^2^ g^−1^ to 4.1 mNm^2^ g^−1^.

#### 2.3.2. Optical Properties

The suberin fatty acid fraction also contained aromatic and chromophoric moieties such as ferulic acid. These compounds gave a brownish color to the SFA fraction resulting in an expected increase of yellowness and a decrease of brightness, as seen in Table 3. The reference sheet yellowness was 51.4%-ISO and increased to 56.6%-ISO by heat treatment alone. The further addition of 10, 20 and 30 gm^−2^ of SFA curing agent increased yellowness to 60.5%, 61.6% and 63.32%-ISO, respectively, and decreased the brightness.

#### 2.3.3. Contact Angle, Air Permeance and Water Vapor Transmission Rate (WVTR)

**Figure 2:**
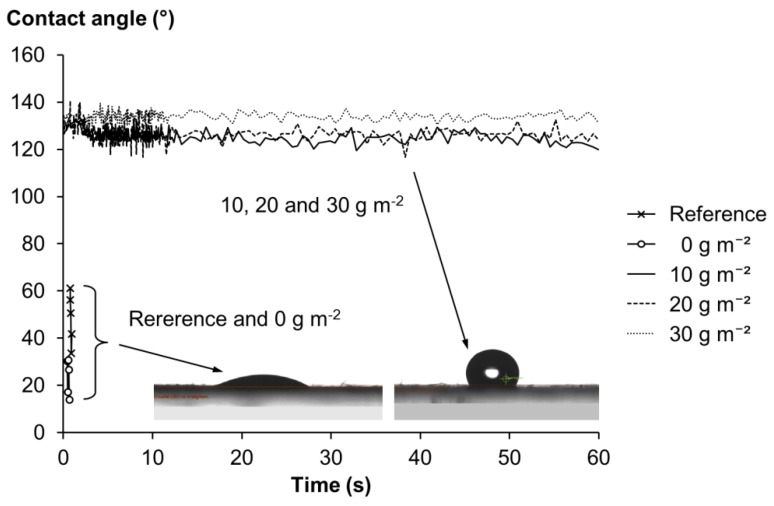
Contact angle measurement of laboratory sheets. Reference is an untreated sheet, 0 g m^−2^ is only heat-treated sheet (150 °C overnight) and 10–30 g m^−2^ is the amount of the added SFAs and curing agent, maleic anhydride. The droplet shape subsequent to deposition on the sheet surface is also visible.

Sheet hydrophobicity was determined with contact angle measurements (Figure 2). The reference sheets and the heat-treated laboratory sheets absorbed completely the water and thus, contact angles could not be recorded. The contact angles of all SFA-treated laboratory sheets were over 120 degrees during 60 s measurement time and the contact angle was correlated to the amount of SFA curing agent. The highest contact angle, approximately 135 degrees, was obtained with 30 g m^−2^ SFA curing agent addition. Similar results were obtained when a suberin monomer, cis-9,10-epoxy-18-hydroxyoctadecanoic acid, isolated from birch outer bark, polymerized by lipase and cured with tartaric acid, was compression molded to cellulose sheets [14]. The contact angles were somewhat lower compared to this study.

Suberin addition and heat treatment of laboratory sheets did not show any effect on air permeance (Table 3). The air permeance values of all sheets were 8820 mL min^−1^, which is the maximum value from the instrument, showing that all the sheets were substantially porous. Also, no major differences were observed in WVTR values. Untreated sheets had slightly lower WVTR values compared to those with SFA curing agent addition, which may be caused by absorbed moisture from the humid air in the measurement chamber.

#### 2.3.4. Scanning Electron Microscope Images and ToF-SIMS

**Figure 3:**
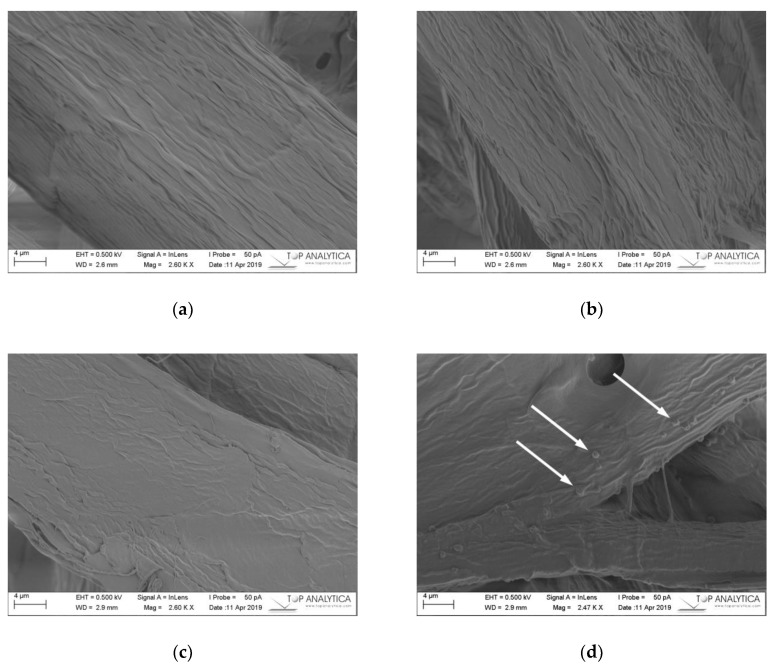
SEM images of fiber surfaces (**a**) the reference handsheet without any treatment (**b**) without curing agent addition but heated at 150 °C overnight (**c**) addition of 20 g m^−2^ curing agent (SFAs and maleic anhydride) and (**d**) addition of 30 g m^−2^ curing agent. Scale bar is 4 µm. Note the droplets of curing agents at higher concentrations (**d**) and arrows.

The microfibrillar structure of the fiber surfaces found in the reference sheet and in the sheet containing 0 g m^−2^ curing agent is clearly visible under electron microscope (Figure 3a–d) resulting in a wrinkled fiber surface. When SFA curing agent was added and the laboratory sheets were heat treated the fiber surfaces were smoothened and microfibrillar structure was not any more visible (Figure 3c,d).

**Figure 4:**
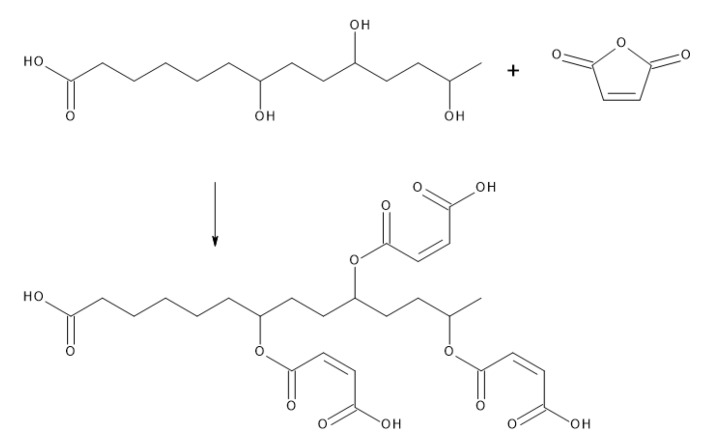
Reaction mechanism of suberin fatty acid monomer with maleic anhydride adapted from Flynn [30] and Mazo et al. [31].

The suberin fatty acid fraction contained hydroxy and epoxy fatty acids (Table 1) and they undergo maleation reaction with maleic anhydrate acting as curing agent, as illustrated in Figure 4.

The maleated SFAs were absorbed by the fibers and filled the voids between the microfibrils. When the SFA curing agent addition was increased to 30 g m^−2^ the same smoothing phenomenon was observed. Additionally, the maleated SFA agent formed globular structures distributed all over the fiber surfaces. It is most likely the fibers were not able to absorb more SFAs and the excess started to precipitate on the surfaces. Also, betulinol and betulinic acid tend to form globular structures on fiber surfaces [8,9]. Both betulinol and betulinic acid were identified in the SFA fraction (Figure 1a and Table 1).

**Figure 5:**
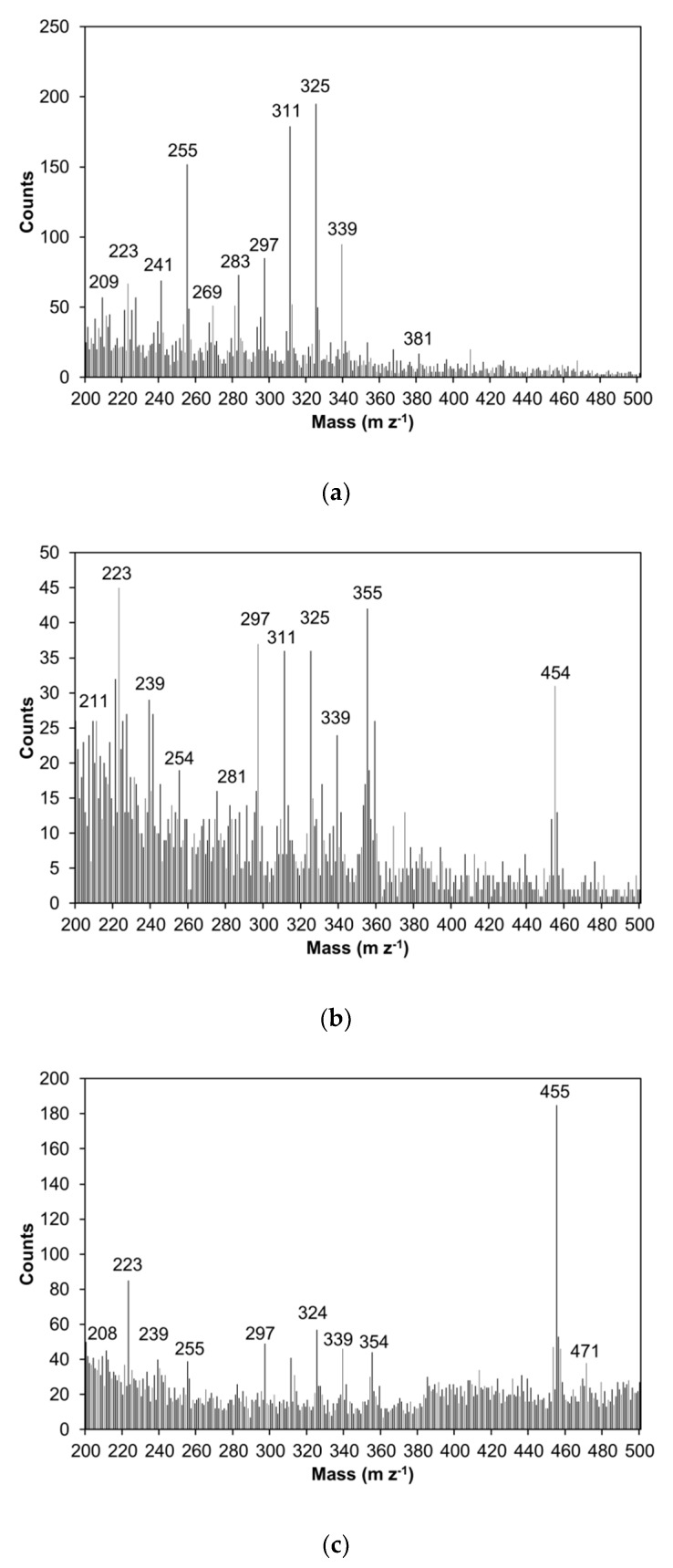
The negative ion ToF-SIMS spectra of the laboratory sheets (**a**) reference handsheet without any treatment (**b**) addition of 20 g m^−2^ curing agent (SFA + maleic anhydride and heat treatment at 150 °C overnight) and (**c**) addition of 30 g m^−2^ curing agent. The peak at 454–455 is betulinic acid.

ToF-SIMS spectrum was taken from the globular structures found in the handsheets with 30 g m^−2^ SFA curing agent addition. Also, other handsheets were analyzed with ToF-SIMS, as seen in Figure 5.

The negative ion ToF-SIMS spectra confirmed the presence of betulin-derived compounds in the fiber surfaces, especially on the globular structures. The peak at the mass-to-charge ratio (m z^−1^) around 456 was relatively intense for the handsheets with SFA curing agent addition (Figure 5b,c). Betulinic acid has molar mass of 456 g mol^−1^. No betulinol-derived compounds were found in the reference sheet. 

## 3. Materials and Methods

Silver birch (*Betula pendula* Roth.) outer bark was manually removed from freshly cut stems (diameter of the trees was 200–300 mm) and air dried. Outer bark was then ground using a cutting mill with a sieve cassette having 4 × 4 mm^2^ square openings. The ground outer bark was then freeze-dried and stored in an airtight polyethylene bag.

Unbleached softwood kraft pulp was obtained from a pulp mill after the blow line. The pulp was washed and screened in a laboratory using a Somerville screen (0.15 mm) to remove shives. The pulp was dewatered after screening and stored in a freezer at −20 °C. No pulp refining (beating) was carried out to obtain as untreated laboratory sheet structure as possible.

Isopropyl alcohol 99.8% (Merck KGaA, Darmstadt, Germany), sodium hydroxide 99.0% (Merck KGaA, Darmstadt, Germany), ethanol 94.0% (Altia Oyj, Rajamäki, Finland) and sulfuric acid 95% (VWR International S.A.S., Briare, France) were used in the experiments. Then, 2 molar sulfuric acid was prepared by pouring 112 mL concentrated sulfuric acid into 600 mL of maxima ultrapure water. The final volume was adjusted with ultra-pure water to 1000 mL after the solution was cooled to room temperature.

### 3.1. Suberin Fatty Acid Extraction and Isolation

**Figure 6:**
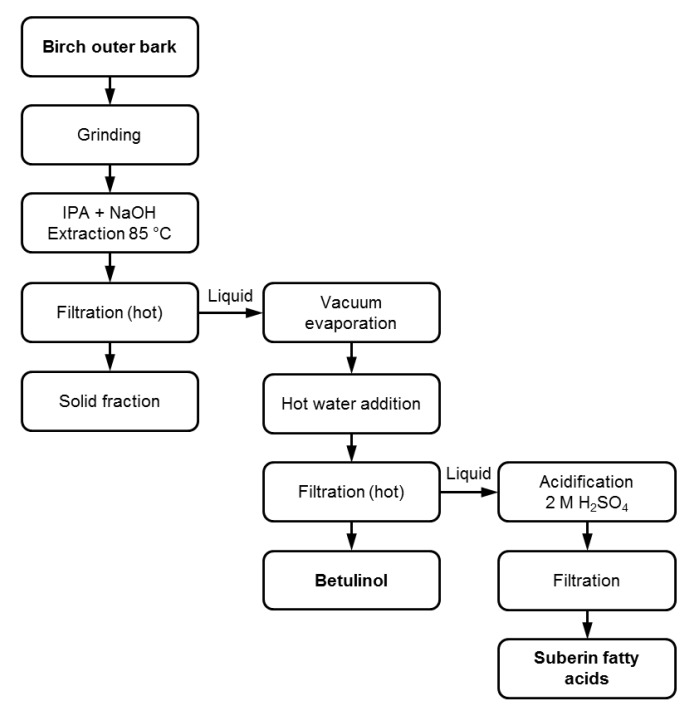
Scheme of the extraction and isolation of suberin fatty acids.

The extraction procedure of suberin fatty acids is shown in Figure 6. The extraction process was adapted from [11,23] but the extraction time was prolonged to three hours.

A total of 100 g oven dry (o.d.) ground bark was placed in a 2000 mL round bottom flask. A volume of 900 mL isopropyl alcohol and 100 mL deionized water was mixed. Sodium hydroxide (20 g) was dissolved in the solution and it was poured in the flask. A reflux condenser was attached and the outer bark was refluxed for three hours. The extract was filtered hot and the bark residue was washed with 500 mL hot isopropyl alcohol and water mixture (9:1 v v^−1^) and filtered again. The two filtrates were combined and cooled to room temperature. The isopropyl alcohol was evaporated under vacuum and 1500 mL hot deionized water was then added. The water-insoluble betulinol fraction was precipitated out and filtered. The solid fraction was washed with 1000 mL hot deionized water and filtered. Again, the two filtrates were combined. The pH value of the combined filtrate containing suberin fatty acid soaps was adjusted to 4.7 using 2 M sulfuric acid. The suberin fatty acids soaps were converted back to fatty acids and the suberin fatty acid fraction was precipitated out from the solution. The suberin fatty acid fraction was washed with 1000 mL deionized water. Both the betulinol and suberin fatty acid fractions were freeze dried and weighed.

### 3.2. Elemental Analysis

Elemental analysis for betulinol and suberin fatty acid fractions was carried out using a closed wet HNO_3_-H_2_O_2_ digestion method (Miller 1998 [32]) in a microwave oven (CEM MDS 2000) and the extract was analyzed by an iCAP 6500 DUO inductively coupled plasma (ICP)-emission spectrometer (Thermo Fisher Scientific, Cambridge, UK).

### 3.3. GC and GC-MS Analysis

The composition of both the betulinol and suberin fatty acids fractions were quantified by gas chromatography flame ionization detection (GC-FID) and the peak identities were confirmed by gas chromatography mass spectrometry (GC-MS). The retention time and mass spectra of birch bark suberin and other low molecular weight components have previously been identified as carboxylic acid methyl esters and silylated alcohols [12,23]. In this analysis the fractions were analyzed as their trimethylsilyl (TMS) esters and ethers. Cholesterol was used as standard. The fractions were silylated with a mixture of *N*,*O*-Bis(trimethylsilyl)trifluoroacetamide (BSTFA): Chlorotrimethylsilane (TMCS): Pyridine (120:20:20) and heated for 50 min at 70 °C. The composition of the fractions by component, their retention time and Kovats’ retention index can be found in Table 1.

The samples were analyzed on a Shimadzu GC-2010 Plus GC (Shimadzu Corporation, Kyoto, Japan) with a flame ionization detector, equipped with an AOC-20i autosampler and a split/splitless injector. The column used was a ZB-1HT (Phenomenex, Torrance, CA, USA), 20 m, 0.18 mm i.d. and film thickness 0.18 µm, coated with 100% polydimethylsiloxane. Initial temperature was 80 °C (1 min), temperature gradient was 8 °C min^−1^ and final temperature was 360 °C (15 min). Injection temperature was 250 °C and detector temperature was 360 °C. Split injection (1 µL) with a ratio of 25:1 was employed. Carrier gas was hydrogen at 40 cm s^−1^ linear velocity.

The samples were also analyzed on a Shimadzu GCMS-QP2010 Plus GC-MS (Shimadzu Corporation, Kyoto, Japan) for component identification. Gas chromatographic conditions were as reported above except for using helium as carrier gas. Mass spectrometer parameters were as follows: interface and ion source temperatures, 345 °C and 230 °C, respectively; ionization mode, electron ionization (EI) with 70 eV; acquisition mass range, 35–800 m z^−1^. Identifications were based on a comparison of the GC retention times (Kovats’ index) and EI spectra with those in our own database.

### 3.4. Preparation of Laboratory Sheets (Lignocellulosic Fiber Network)

Laboratory sheets were prepared in a standard sheet former with 60 g m^−2^ target grammage. Wet sheets were pressed twice, first 5 min at 400 kPa, then 2 min at 400 kPa. The sheets were dried in conditioned air (23 °C temperature and 50% relative humidity) using drying plates. Dried sheets were cut to squares having a 0.02 m^2^ area and stored in aluminum foil.

### 3.5. Suberin Fatty Acid Impregnation and Curing

A curing agent solution containing 50 mg ml^−1^ suberin fatty acids and 50 mg ml^−1^ maleic anhydride in ethanol was prepared. Dry solids of 10, 20 and 30 g m^−2^ (2.0, 4.0 and 6.0 mL curing agent) were applied on the laboratory sheets and the laboratory sheets were placed on an aluminum foil. The dosages were selected on the basis of commercial polymer coating grammage of paperboard [33,34]. The volume of ethanol in 10 g m^−2^ was so small that 2 mL of additional ethanol was used in order to distribute suberin fatty acids and maleic anhydride evenly in the laboratory sheets. The solvent was evaporated in an oven at 70 °C for 30 min and the laboratory sheets were removed from the aluminum foil. The temperature was then increased to 150 °C and the laboratory sheets were kept in the oven overnight. One reference set of laboratory sheets was only heat treated (0 g m^−2^) and was composed only of pure fibers (pure laboratory sheet).

### 3.6. Paper Technical Properties

Thickness, grammage, tensile strength, tear strength, brightness, yellowness and air permeability were measured for the laboratory sheets according to the following ISO standards:

- Thickness, density, grammage: ISO 534 Paper and board—Determination of thickness, density and specific volume.
- Tensile strength: ISO 1924 Paper and board—Determination of tensile strength.
- Tear strength: ISO 1974 Paper—Determination of tearing resistance.
- Optical properties: ISO 2469 Paper, board and pulps—Measurement of diffuse radiance factor (diffuse reflectance factor) and ISO 2470-1 Paper, board and pulps—Measurement of diffuse blue reflectance factor—Part 1: Indoor daylight conditions (ISO brightness).
- Air permeance: ISO 5636 Paper and board—Determination of air permeance and air resistance (medium range).

### 3.7. Water Vapor Transmission Rate

The water vapor transmission rate (WVTR) was determined, using the relative humidity (RH) gradient of 0%/54%. Fiber sheets were sealed on aluminum cups containing 43 g CaCl_2_ as a desiccant, with the top side of the laboratory sheet facing up towards the moist side. There was an air gap of 6 mm width between the salt and the wire side of the laboratory sheet. The cups were placed in a desiccator cabinet equipped with a fan to circulate the air above the samples at a speed of 0.15 m s^−1^. The cabinet was kept at constant temperature of 24 °C and the RH was maintained at 54% using saturated Mg(NO_3_)_2_ solution. The cups were weighed after 0, 115, 1120, 1340 and 1555 min. The temperature and the RH of the cabinet were measured using a Rotronic RH meter (Rotronic AG, Bassersdorf, Switzerland) before each weighing. The water vapor transmission rate was calculated from the linear regression of the slope of weight gain vs. time by dividing the slope by the treated laboratory sheet area. Three replicates of each paperboard type were tested. The thickness of the specimens was measured before testing at five points with a micrometer (Lorentzen & Wettre, Kista, Sweden, precision 1 µm).

### 3.8. Contact Angle Measurement

The contact angles of ultrapure water on the coated paperboards were measured using a CAM 200 Series Optical Contact Angle and Surface Tension meter (KSV Instruments Ltd.; Helsinki, Finland, now part of Biolin Scientific, Stockholm, Sweden). The drop size was set to 4 µl and three parallel measurements were performed for each substrate. The contact angle was calculated as an average of the right and left angles 1 s after the drop was detached. The measurement time was 60 s.

### 3.9. Scanning Electron Microscopy

Scanning electron microscopy (SEM) images were obtained by Zeiss GeminiSEM 450 field emission scanning electron microscope (Carl Zeiss Microscopy GmbH, Jena, Germany) equipped with secondary electron analyzer. Acceleration voltage was 0.500 kV and probe current 50 pA. Gold sputtering was applied on the samples prior to imaging.

### 3.10. Time-of-Flight Secondary-Ion Mass Spectrometry

Secondary ion mass spectra were obtained using a Physical Electronics ToF-SIMS TRIFT II spectrometer (Physical Electronics Inc., Chanhassen, MN, USA). A primary ion beam of 69 Ga^+^ liquid metal ion source (LIMS) with 25 kV accelerating voltage and 600 pA beam current (in DC mode) was used in both positive and negative modes. The measurements were done from an area 200 × 200 µm and the analysis depth is in the order of few nanometers. The measurement time of 5 min was used and the total ion dose was <1012 ions cm^−2^. Charge compensation was obtained with an electron flood gun pulsed out of phase with respect to the ion gun.

## 4. Conclusions

Suberin fatty acids obtained from birch outer bark, a renewable resource, can be utilized when creating fibrous materials with excellent water repellent properties. Laboratory sheets with only 10 g m^−2^ of SFA curing agent treatment already showed hydrophobicity. Suberin fatty acids were evenly distributed on the fiber surfaces covering microfibrillar structures after curing. The larger amounts of SFA resulted in globular structures on the fiber surfaces further enhancing the hydrophobicity. The SFA curing agent treatment impaired some laboratory sheet properties such as tear strength and brightness. However, a slight change in color is negligible in packaging materials made of unbleached fibers because they are already brown. The SFA curing agent treatment improved tensile strength, an important property for packaging materials.

## Figures and Tables

**Table 1:** The composition of suberin fatty acid (SFA) and betulinol fractions from birch outer bark. Expressed as mg g^−1^ (o.d.) weight. Retention times and Kovats’ retention indices are also visible.

Compound	SFA Fraction (mg g^−1^)	Betulinol Fraction (mg g^−1^)	Retention Time (min)	Kovats’ RI
Ferulic acid	1.0		15.765	2073
17:0 fatty acid (margaric acid)	0.1		16.537	2141
18:2 fatty acid (linoleic acid)	1.0		17.153	2200
18:1 fatty acid (oleic acid)	0.4		17.254	2208
18:0 fatty acid (stearic acid)	0.2		17.600	2239
16-hydroxy-16:0 acid	2.7		19.601	2395
20:0 - fatty acid (arachidic acid)	0.5		19.208	2437
1,16-dioic-16:0 acid	3.5		19.957	2472
18:-hydroxy-(9)18:1 acid	62.4	2.0	20.778	2558
9,16- and 10,16-dihydroxy-16:0 acids	19.7		21.032	2580
18-hydroxy-18:0 acid	1.5		21.087	2591
1,18-dioic-(9)18:1 acid	16.8		21.448	2633
1,18-dioic-18:0 acid	5.0		21.786	2669
9,18-dihydroxy-(9)18:1 acid	5.0		22.202	2713
9,10-epoxy-18-hydroxy-18:0 acid	198.0	2.3	22.496	2746
20-hydroxy-20:1 acid	6.2		22.536	2752
Dihydroxyoctadecanoic acid	4.4		22.721	2769
20-hydroxy-20:0 acid	16.5	0.9	22.852	2787
1,20-dioic-20:1 acid	2.4		23.157	2828
24:0 fatty acid (lignoceric acid)	0.4		23.210	2834
1,20-dioic-20:0 acid	6.0		23.497	2866
9,10,18-trihydroxy-18:0 acid	70.2	0.6	23.769	2888
22-hydroxy-22:0 acid	94.1	6.6	24.541	2988
1,22-dioic-22:0 acid	7.7		25.116	3064
Cholesterol (standard)	-	-	25.592	3151
24-hydroxy-24:0 acid	2.8		25.990	3178
Lupenone (lup-20(29)-en-3-one)	0.5	1.1	26.866	3330
Sitosterol	1.3	2.3	27.010	3347
Lupeol	3.8	50.8	27.310	3397
Betulonic acid		2.4	28.231	3529
Betulinol	16.8	802.4	28.628	3574
Betulinic acid	53.1	23.8	28.799	3599
Monogynol A (lupane-3b,20-diol)	0.0	6.1	29.478	3706
Lupane-3b,20,28-triol	0.0	2.3	29.950	3766
Total identified	607.2	901.2		
Total eluted	743.7	971.8		

**Table 2:** Elemental analysis of the obtained suberin fatty acid and betulinol fractions. Elements are listed in decreasing order.

SFA Fraction		Betulinol Fraction	
Element	Content (mg kg^−1^)	Element	Content (mg kg^−1^)
S	210	Na	4910
Na	77.2	Mn	42.3
P	29.8	Mg	22.7
K	<10.2	S	22.1
Ca	3.83	Fe	18.8
Pb	<1.02	Ca	17.7
Cu	0.782	K	17
Al	0.671	P	12.8
B	0.508	Zn	2.56
Fe	0.335	Al	2.38
Zn	0.305	Pb	<1.02
Mg	0.213	B	0.969
Cr	<0.203	Cu	0.948
Ni	<0.203	Cr	<0.204
Cd	<0.07	Ni	<0.204
Mn	0.061	Cd	<0.07

**Table 3:** Properties of laboratory sheets. Reference is untreated sheet, 0 g m^−2^ is only heat treated (150 °C overnight) and 10–30 g m^−2^ is the amount of added curing agent (SFA + maleic anhydride) and heat treatment. The standard deviations, if applicable, are in the parenthesis.

	Grammage	Thickness	Density	Tear Index	Tensile Index	Brightness	Yellowness	WVTR	Air Permeance
	(g m^−2^)	(µm)	(kg m^−3^)	(mNm^2^ g^−1^)	(Nm g^−1^)	(%-ISO)	(%-ISO)	(g m^−2^d^−2^)	(ml min^−1^)
Reference	56.3	183.5 (8.1)	306.7 (13.5)	5.7 (0.4)	11.5 (0.9)	26.1 (0.3)	51.4 (0.2)	2523 (113)	8820
0 g m^−2^	57.9	181.0 (6.0)	319.9 (10.4)	5.7 (0.2)	14.0 (0.6)	24.8 (0.1)	56.6 (0.2)	2577 (11)	8820
10 g m^−2^	62.3	190.9 (8.4)	326.4 (15.0)	4.6 (0.2)	16.6 (0.8)	24.4 (0.1)	60.5 (0.2)	2576 (78)	8820
20 g m^−2^	66.9	204.8 (4.6)	326.8 (7.5)	4.2 (0.1)	16.7 (1.8)	23.9 (0.3)	61.6 (0.6)	2755 (29)	8820
30 g m^−2^	69.8	207.4 (2.8)	336.7 (4.6)	4.1 (0.4)	16.0 (4.1)	22.5 (1.9)	63.2 (3.5)	2847 (47)	8820

## Abbreviations

The following abbreviations are used in the manuscript:

BSTFA	*N*,*O*-Bis(trimethylsilyl)trifluoroacetamide
EI	Electron ionization
FID	Flame ionization detection
GC	Gas chromatography
ICP	Inductively coupled plasma
LIMS	Liquid metal ion source
MS	Mass spectrometry
o.d.	Oven dried
RH	Relative humidity
SEM	Scanning electron microscopy
SFA	Suberin fatty acid
TMCS	Chlorotrimethylsilane
TMS	Trimethylsilyl
ToF-SIMS	Time-of-flight secondary ion mass spectrometry
WVTR	Water vapor transmission rate
